# Transcript and protein signatures derived from shared molecular interactions across cancers are associated with mortality

**DOI:** 10.1186/s12967-024-05268-7

**Published:** 2024-05-11

**Authors:** Yelin Zhao, Xinxiu Li, Joseph Loscalzo, Martin Smelik, Oleg Sysoev, Yunzhang Wang, A. K. M. Firoj Mahmud, Dina Mansour Aly, Mikael Benson

**Affiliations:** 1 Medical Digital Twin Research Group, Department of Clinical Science, Intervention and Technology (CLINTEC), Karolinska Institutet, Stockholm, Sweden; 2 Channing Division of Network Medicine, Department of Medicine, Brigham and Women’s Hospital, Harvard Medical School, Boston, MA USA; 3 Division of Statistics and Machine Learning, Department of Computer and Information Science, Linköping University, Linköping, Sweden; 4 Department of Clinical Sciences, Danderyd Hospital, Karolinska Institutet, Stockholm, Sweden

**Keywords:** Cell–cell interactions, Cancer-associated fibroblast, Single-cell RNA sequencing, Prioritization, Pan-cancer, Mortality

## Abstract

**Background:**

Characterization of shared cancer mechanisms have been proposed to improve therapy strategies and prognosis. Here, we aimed to identify shared cell–cell interactions (CCIs) within the tumor microenvironment across multiple solid cancers and assess their association with cancer mortality.

**Methods:**

CCIs of each cancer were identified by NicheNet analysis of single-cell RNA sequencing data from breast, colon, liver, lung, and ovarian cancers. These CCIs were used to construct a shared multi-cellular tumor model (shared-MCTM) representing common CCIs across cancers. A gene signature was identified from the shared-MCTM and tested on the mRNA and protein level in two large independent cohorts: The Cancer Genome Atlas (TCGA, 9185 tumor samples and 727 controls across 22 cancers) and UK biobank (UKBB, 10,384 cancer patients and 5063 controls with proteomics data across 17 cancers). Cox proportional hazards models were used to evaluate the association of the signature with 10-year all-cause mortality, including sex-specific analysis.

**Results:**

A shared-MCTM was derived from five individual cancers. A shared gene signature was extracted from this shared-MCTM and the most prominent regulatory cell type, matrix cancer-associated fibroblast (mCAF). The signature exhibited significant expression changes in multiple cancers compared to controls at both mRNA and protein levels in two independent cohorts. Importantly, it was significantly associated with mortality in cancer patients in both cohorts. The highest hazard ratios were observed for brain cancer in TCGA (HR [95%CI] = 6.90[4.64–10.25]) and ovarian cancer in UKBB (5.53[2.08–8.80]). Sex-specific analysis revealed distinct risks, with a higher mortality risk associated with the protein signature score in males (2.41[1.97–2.96]) compared to females (1.84[1.44–2.37]).

**Conclusion:**

We identified a gene signature from a comprehensive shared-MCTM representing common CCIs across different cancers and revealed the regulatory role of mCAF in the tumor microenvironment. The pathogenic relevance of the gene signature was supported by differential expression and association with mortality on both mRNA and protein levels in two independent cohorts.

**Supplementary Information:**

The online version contains supplementary material available at 10.1186/s12967-024-05268-7.

## Introduction

According to the WHO global cancer cases will increase by more than 75% by 2050, significantly increasing mortality [1]. This increase involves highly diverse cancers in both women and men. Could this indicate that, despite this diversity, there are shared mechanisms across cancers? If so, are those mechanisms important for pathogenesis and mortality?

Previous studies of highly diverse complex disease have shown shared mechanisms despite great complexity and heterogeneity. In support of their pathogenic importance those mechanisms are highly interconnected, and enriched for disease-associated genetic variants, so that their combined effects are large [2]. The existence of shared genes across cancers is supported by a previous study of deconvoluted bulk RNA sequencing data from 20 solid tumor types, which found converging molecular interactions between cancer and stromal cells in the tumor microenvironment (TME) [3]. A limitation of this study was that the deconvoluted data did not yield the cellular resolution of single cell data, so that potentially important cell types were not included in the analyses. For example, this study primarily investigated cancer and stromal cells, overlooking the diverse functions of individual stromal cell types like fibroblasts and endothelial cells. Moreover, immune cells were not studied, despite their important roles in the TME. Another study focused on cell–cell interactions (CCIs) between fibroblast subtypes and tumor cells in six different cancers and found associations with the response to immunotherapy [4]. However, this article also did not systematically evaluate CCIs across all cell types. While Scherz-Shouval’s group specifically characterized molecular interactions between tumor cells and many other cell types in the TME in breast cancer, it was focused on one cancer rather than shared mechanisms across cancers [5]. Moreover, the clinical implications of their findings were not evaluated in a pan-cancer setting in the aforementioned studies.

These pioneering studies supported the idea that there may be shared ligand–target interactions between specific cell types across cancers. If so, such interactions could have important implications: Since carcinogenesis involves multiple cell types, and not only malignant ones, CCIs could constitute a higher order representation of the complex and heterogeneous changes in all those cell types [6]. However, these studies limited the analysis to two to three cell types. This leads to an unanswered question: Are there shared CCIs when all cell types in different tumors are analyzed? If so, would it be possible to systematically organize those into one comprehensive model, which could be used to prioritize the most important interactions? Previous single-cell RNA sequencing (scRNA-seq) studies of inflammatory diseases have used such CCIs to construct multicellular models. In those models, the upstream regulatory (UR) ligands could be ranked and prioritized based on the relative numbers of cell types and downstream target (DS) genes. The disease relevance of the models and URs was validated by functional studies [7–9]. Here, we translated the same principles to construct multicellular tumor models (MCTMs) of different cancers based on scRNA-seq data. We next hypothesized that those MCTMs could be used to construct a shared MCTM (shared-MCTM) from which a shared gene signature could be prioritized. This did result in the identification of an shared-MCTM and a gene signature, whose pathogenic relevance was validated by differential mRNA and protein expression, as well as association with mortality in independent data from The Cancer Genome Atlas (TCGA, 9185 tumor tissues, 727 control tissues from cancers of 22 different tissue origins) and from the UK Biobank cohort (UKBB, 10,384 cancer patients, 5063 controls with proteomics data of cancers from 17 different tissue origins).

## Methods

### Data source

#### ScRNA-seq

ScRNA-seq count matrix files of five common cancers: breast cancer (ER-positive breast cancer, GSE161529 [10]), colon cancer (colorectal cancer, GSE144735 [11]), liver cancer (intrahepatic cholangiocarcinoma, GSE138709 [12]), lung cancer (lung adenocarcinoma, GSE123902 [13] and ovarian cancer (E-MTAB-8107 [14]) from Gene Expression Omnibus (GEO) [15] or ArrayExpress [16] (Additional file 1 S1). These cancer datasets were selected because they are among the most prevalent solid tumors, and high-quality scRNA-seq data of untreated primary tumors and control samples (adjacent normal tissue, except for the breast cancer dataset, where normal tissues were from mammary gland cells of non-breast cancer patients) were available. All retrieved scRNA-seq studies were performed using 10 × Genomics’ scRNA-seq technology.

#### UK biobank

Proteomic data from the UKBB cohort includes plasma proteome of 54,306 unique UKBB participants from the UK Biobank Pharma Proteomics Project [17]. The expression of 2911 plasma proteins (the second release) were tested using the antibody-based Proximity Extension Assay by Olink and was provided as Normalized Protein Expression (NPX) [17]. NPX is a relative quantification unit related to protein concentration; it was background-corrected, log2-transformed, and normalized within all samples [18]. The identification of cancer cases and healthy controls was performed using the International Classification of Diseases (ICD) coding system, specifically ICD-9 and ICD-10 (Additional file 1 S2). A detailed description of the UKBB proteomic data and the sample size is provided in the Supplemental Methods and Additional file 1 S3. Proteins with more than 20% missing data were removed, and the one with less than 20% missing were imputed using the K-nearest neighbor (KNN) method with 10 nearest neighbors.

#### TCGA and GTEx

The mRNA transcripts per million (TPM) expression profiles of tissues from 22 organ sites were obtained from TCGA and GTEx through UCSC Xena [19]. The gene expression was transformed to log2(TPM + 0.001) for both TCGA and GTEx samples by the same RNA-seq pipeline^.^ The normal sample of GTEx was combined with the normal sample in TCGA according to organ site, the merging strategy and sample size for each dataset are listed at Additional file 1 S4.

### Ethics

The UKBB study received ethical approval from the National Information Governance Board for Health and Social Care and the National Health Service Northwest Multi-Center Research Ethics Committee, and all participants provided written consent. This research has been conducted under approved application number 102162. All participants in TCGA were consented, and the data is openly accessible to researchers.

### ScRNA-seq processing

The downloaded count matrices of each cancer scRNA-seq data set were processed and quality controlled using the R package Seurat v4.0.4 [20]. For each sample, the low-quality cells were filtered out based on mitochondrial RNA percentage, the range of read counts, and gene coverage (Supplement Methods). Each cancer was analyzed independently. Single-cell profiles from different samples within the same cancer were integrated using Seurat 4 anchor-based integration methods *IntegrateData*. Cell clusters were identified using the default *FindClusters* function. Cell types were annotated by known cell type markers detailed in the Additional file 2. A Model-based Analysis of Single-cell Transcriptomics (MAST) [21] was used for the identification of differentially expressed genes (DEG) between tumor tissues and normal tissues within the same cell type. DEGs with adjusted p-value < 0.05 and absolute log2 transformed fold change (log2FC) > 0.25 were used for downstream analysis. For each cell type, a positive log2FC indicated upregulation in tumor tissue compared to normal tissue while a negative value indicated downregulation in tumor tissues.

Fibroblasts from each cancer were extracted and integrated into one dataset using the Seurat *IntegrateData* function to adjust the difference between cancers. The clustering resolution was 0.1 with seed 42. Marker genes of each cluster were identified using the *FindAllMarkers* function with default settings. Similar analyses were performed for epithelial and endothelial cells.

### Construction of MCTM and shared-MCTM

To infer cell–cell interactions (CCIs) of all cell type pairs, an R package NicheNet (v1.1.0) was applied [22]. This analysis was performed separately for each cancer. In brief, the cell type and DEGs list for each cell type served as input for NicheNet. CCIs were then identified between each pair of cell types using the default analysis setup. For each identified CCI, potential upstream regulatory (UR) ligands and downstream target (DS) genes in the source and target cell type were determined using the *predict_ligand_activities* and *get_weighted_ligand_target_links* functions with default settings. The predicted interactions for all cell type pairs in each cancer were used to construct a MCTM. The MCTM, thus, consists of cell types as nodes and cell–cell interactions as edges. The edge weight was proportional to the number of interactions between the two cell types. All URs and DSs used to construct MCTM were referred to as MCTM genes.

To identify common CCIs across all five cancers, a shared-MCTM was created as follows: (1) URs found in all cancers were identified. (2) For each cell type, the log2FC of each UR from step 1 was compared among all five cancers. A UR was considered a shared UR (shared-UR) if it exhibited the same direction of expression change in one cell type in at least four cancers. The cell type of this UR was recorded and used for shared-MCTM construction. (3) Subsequently, DSs of shared-URs were identified; we defined shared DSs (shared-DSs) using the same criteria applied for identifying shared-URs. (4) The genes (shared-URs and shared-DSs) and their corresponding cell types (shared-MCTM cell types) were used for constructing the shared-MCTM.

### Prioritization of shared-URs

To systematically prioritize shared-URs, shared-URs were clustered based on the number of interactions with each downstream cell type in the shared-MCTM. Euclidean distance was used for clustering and clusters were cut into two main subclusters according to the dendrogram. The cluster with a larger number of interactions in all cell types was considered as the top cluster, and shared-URs in this cluster were considered as top shared-URs.

### Genome-wide association studies (GWAS) gene enrichment analyses and disease relevance

GWAS gene enrichment analysis (Fisher’s exact test, double-sided) of MCTM genes was performed for each cell type/cancer separately. All DEGs identified in that cell type/cancer were used as a background. The null hypothesis is that, compared to all DEGs in each cell type/cancer, there is no association between the MCTM genes in this cell type/cancer and GWAS-associated genes. GWAS gene enrichment was also performed for shared-MCTM genes. For each cancer, the null hypothesis is that compared to all DEGs found in each cancer, there is no association between the shared-MCTM genes and GWAS-associated genes for this cancer type. The FDR method was applied for multiple comparisons adjustment and an adjusted p-value < 0.05 indicated a significant enrichment of GWAS genes in MCTM or shared-MCTM. The GWAS-associated genes were downloaded from DisGeNET in November 2021 [23]. The “diseaseName” and GWAS genes for each cancer were listed in Additional file 2 S2.

The disease relevance was computed using DisGeNET “disgenet2r” R package version 0.99.2. To perform disease enrichment of genes included in the shared-MCTM, default setting of the *disease_enrichment* function was used. The p-values resulting from the multiple Fisher tests were corrected for multiple testing using the False Discovery Rate (FDR) method.

### KEGG enrichment

KEGG enrichment was performed using the R clusterProfiler package (v3.18.1) [24]. The KEGG enrichment for marker genes of each cancer-associated fibroblast (CAF) subcluster was performed using function *enrichKEGG*. Function *compareCluster* was used for plotting the top KEGG terms of shared-DSs of shared-URs expressed in fibroblast shared-Urs.

### Definition of all-cause mortality and survival time

The all-cause mortality was defined as death with any reason during the observation period (10 years after cancer diagnosis). The survival time was defined as the period from initial cancer diagnosis until the date of death from any cause, loss to follow-up or the end of the follow-up period (30 November 2022 in UKBB) [25].

### Gene set and protein set scoring

The gene score of signature genes was calculated for cancer patients in TCGA. The default “gsva” method in the GSVA R package was used for calculating these scores [26]. The corresponding protein score was calculated for the UKBB cancer patients using the average NPX of proteins encoded by signature genes. The gene score and protein score were divided into high and low groups using their average value as cutoff.

### Statistics

Differential expressions of mRNAs and proteins were tested between tumor tissue vs. normal tissue or cancer patient vs. healthy control in TCGA or UKBB, respectively. The differential expression of each mRNA or protein was assessed for each individual cancer using the two-sided Wilcoxon test, and the difference of expression was presented as log2FC.

The survival analysis was performed in all cancer patients pooled together and each cancer individually. The Cox proportional hazards model was used to calculate hazard ratios (HRs) and 95% confidence intervals (Cis) for the associations of each mRNA or protein (and the mRNA signature score or protein signature score) with the 10-year mortality of patients who diagnosed cancer. This association was also performed in each sex subgroup. The Cox models were adjusted for basic confounding factors when appropriate (UKBB: sex, age of diagnosis, time difference from diagnosis to sampling, and cancer type; TCGA: sex, age of diagnosis, and cancer type). Sex was excluded from the model when performing survival analysis in each sex subgroup, and cancer type was excluded from the model when testing in each individual cancer. Cancers with less than 20 death events were excluded when testing the association in each individual cancer. Kaplan–Meier survival curves were plotted for the combination of signature score level (high or low) and sex (female or male) using the *ggsurvplot* function and compared using the two-sided log-rank test. All statistical analyses were performed using R (version 4.0.4). The FDR method was applied for multiple comparisons, and an adjusted p-value < 0.05 indicated a significant difference.

## Results

### Overall design

**Fig. 1:**
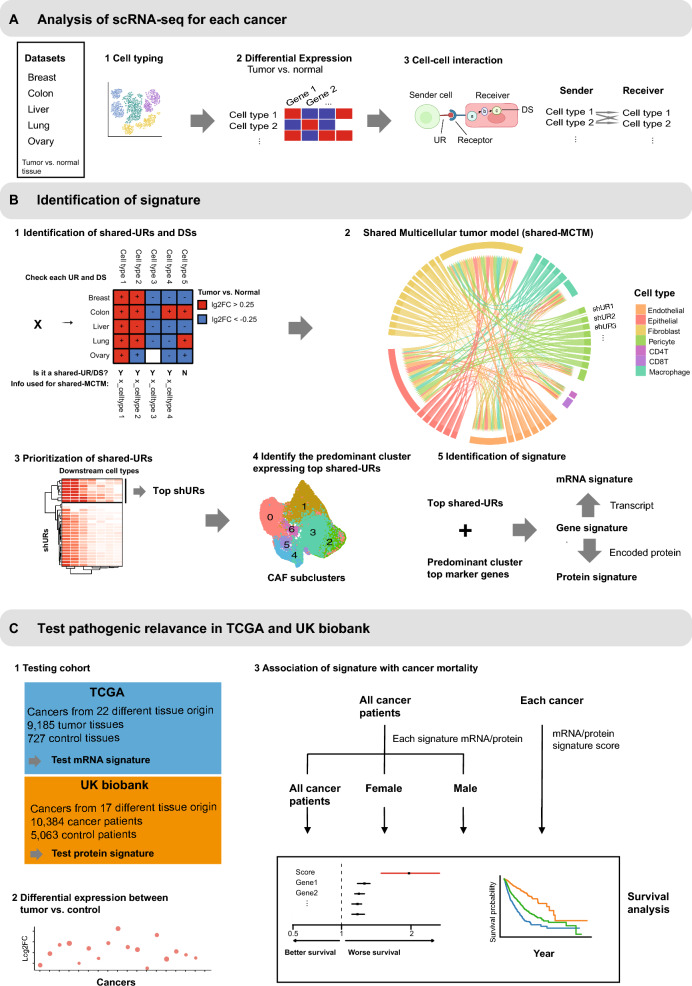
Overview of the study. **A** Single cell and cell–cell interaction (CCI) analyses of each cancer dataset separately. A1 and A2, clustering, cell typing, and differentially expressed genes (DEG) identification. A3, identification of CCIs using DEGs for each cell type pair. The sender/receiver cell type and UR/DS were used to construct MCTM for each cancer. **B** B1, schematic figure depicting how shared upstream regulator gene (shared-URs) and shared downstream target genes (shared-DSs) were identified. For each UR and DS identified by NicheNet, the fold change between tumor vs. normal was examined within each cell type that the identified shared-URs, shared-DSs and the four cell types were connected to construct the shared-MCTM. Red and blue denote increased and decreased expression in the tumor, respectively, while white means no difference. B2, A shared-MCTM representing shared CCIs was constructed using shared-URs, shared-DSs, and their interactions. Each color of the outer ring represents one cell type, which is connected by predicted molecular interactions, the directions of which are indicated by pointed curved lines. B3, shared-URs were prioritized based on the numbers of shared-DSs and cell types. B4, identification of the predominant cluster expressing top shared-URs. B5, top shared-URs and the top marker genes of the predominant cluster were combined to a gene signature with the concordant mRNA and protein signatures. **C** The pathogenic relevance of the mRNA/protein signatures were tested in The Cancer Genome Atlas (TCGA) and UK biobank (UKBB). C1, description of the two testing cohorts. C2, mRNA/protein expression differences between tumor vs. normal in both cohorts. C3, the associations of mRNA/protein signatures with 10-year all-cause mortality in cancer patients from both cohorts

Our hypotheses were that (1) there were shared cell–cell interactions (CCIs) across cancers and that (2) these interactions were important for pathogenesis and mortality. To test the first hypothesis, we analyzed single-cell datasets from different cancers and compared the CCIs between them. This resulted in a shared-MCTM that represented shared cellular interactions across different cancers, from which we identified a gene signature that consisted of prioritized genes (Fig. 1A and B). For the second hypothesis, we assessed the signature at both mRNA and protein levels, subsequently referred to as the mRNA signature and protein signature, in two extensive independent cohorts (TCGA and UKBB). We first compared the expression differences of signature mRNAs between tumor and control: Next, we tested the association of the mRNA/protein signatures with 10-year all-cause mortality in cancer patients (Fig. 1C).

### Analyses of scRNA-seq data from five different cancers shows shared differentially expressed genes

**Fig. 2:**
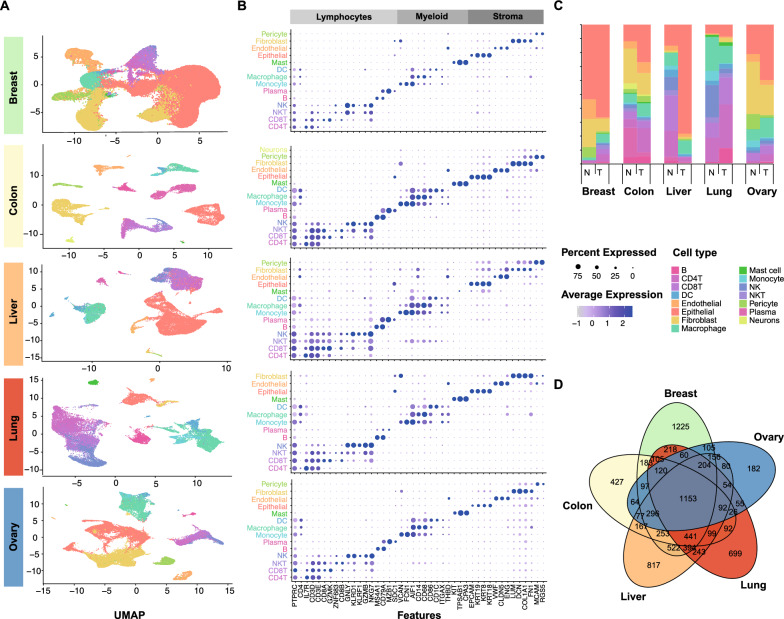
Cellular and molecular heterogeneity of cancer. **A** Clustering of each cancer, colored by cell type. **B** Expression of known marker genes of each cell type in each cancer. **C** Proportion of cell types in each cancer. **D** The overlap of DEGs of each cancer

Given the importance of multiple local cell types other than tumor cells (e.g. stromal cells and immune cells) we conducted analysis of scRNA-seq data from five common cancers, namely breast, colon, liver, lung, and ovarian cancers. Following quality control procedures, a total of 281,302 cells was analyzed and clustered (Fig. 2A). For each cancer, 12–15 distinct cell types were identified, with the expression of known cell type marker genes illustrated in Fig. 2B. The proportions of cell types in the tumor microenvironment differed greatly between the five cancers (Fig. 2C). Epithelial cells and fibroblasts predominated in breast, ovary, colon, and liver cancers, while immune cells were more prevalent in lung cancer. Across all five cancers, the proportion of epithelial cells increased in tumor tissue. Liver cancer exhibited a significant increase in epithelial cells but a decreased proportion of immune cells. These changes in cellular proportions were associated with thousands of DEGs between tumor and normal tissue, which also varied greatly between cell types and cancers (Additional file 3). Nevertheless, we identified 1153 DEGs that were shared across these five cancers (Fig. 2D). This led us to ask if these DEGs were associated with shared interactions between the cell types in the cancers.

### Multi-cellular tumor models show dispersion of pathogenic mechanisms

To search for shared interactions, we first constructed MCTMs of each of the five cancers. The MCTMs were constructed using the CCIs inferred by NicheNet between each cell type pair (sender/receiver cell types and URs/DSs). Therefore, each MCTM showed directed molecular interactions between URs in any cell type and DSs in other cell types (Additional figure S1A). The median (range) number of URs per cancer was 203 (155–232), with 74 URs found in all five cancers (Additional figure S1B and Additional file 4). The median (range) number of DSs per cancer was 1641 (1279–2135), with 577 shared across all cancers (Additional figure S1C and Additional file 4). Rather than a hierarchical organization in which most interactions originated from cancer cells, the interactions formed highly interconnected networks (Additional figure S2 and Additional file 4). Compared to all DEGs in tumor tissue, the MCTM genes in most cell types were enriched with cancer related traits identified by GWAS (Additional figure S3). This suggested that pathogenic mechanisms were distributed across cell types rather than originating solely from cancer cells.

### Construction of a shared MCTM

**Fig. 3:**
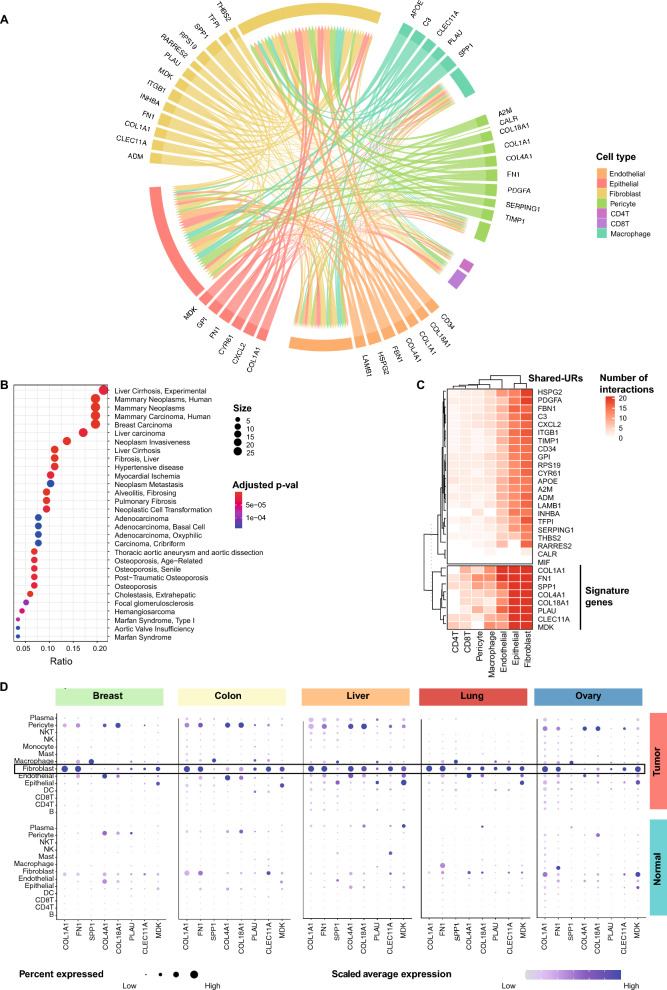
Analyses of a shared-MCTM. **A** A shared-MCTM that represented the shared CCIs across five cancers (the construction principles are outlined in Fig. 1). Each edge shows the shared-UR and its predicted, directed interaction towards its downstream cell type; the thickness of each edge represents the number of shared-DSs; the color of the edges indicates the cellular origin of each interaction. **B** The disease relevance of the shared-MCTM genes was supported by pathway analyses in DisGeNET. Size indicates the number of genes that were enriched in each term and color indicates the significance level after FDR adjustment. **C** shared-URs were clustered based on their predicted downstream effects. Red spectra show the total number of interactions of each shared-UR towards its shared-DSs in each downstream cell type. White indicates no downstream genes shared-URs with larger downstream effects were selected for a gene signature representing the shared-MCTM. **D** Expression of prioritized shared-URs in cell types from tumor and normal tissues. The dot size indicates the percent expression in each cell type and the color scale indicates the expression level

To identify potential shared interactions across cancers, we explored the possibility of constructing a shared-MCTM from the five MCTMs. To characterize interactions, we identified URs and DSs that were shared across the MCTMs (shared-URs and shared-DSs). The criteria for shared-URs and shared-DSs were that they should (1) be URs or DSs in all five cancer MCTMs, and (2) have the same direction of expression change in the same cell type in at least four cancers (Fig. 1B). A total of 117 shared-MCTM genes (30 shared-URs and 98 shared-DSs) located in shared-MCTM cell types (fibroblast, cancer cells, macrophages, endothelial cells, pericytes and T cells) were identified and used to construct the shared-MCTM (Fig. 3A and Additional file 5).

In support of the pathogenic relevance of shared-MCTM, the shared-MCTM genes (shared-URs and shared-DSs) exhibited enrichment for GWAS-associated genes in the five studied cancers from the DisGeNET database, with odds ratios ranging from 2.51 to 3.81 (adjusted p-value < 0.05, except for 0.06 in Ovarian cancer, Additional file 5). Additionally, the shared-MCTM genes were found to be associated with malignant and fibrotic diseases, as indicated by the DisGeNET database (Fig. 3B). These observations underscored the pathogenic importance not only of malignant cells but also of fibroblasts relative to other cell types in the TME.

### Prioritization of a shared gene signature based on the shared-MCTM

To prioritize a shared gene signature based on the shared-MCTM, we focused on the shared-URs that regulated the largest number of shared-DSs. Briefly, we clustered the shared-URs based on their total number of interactions towards each downstream cell type in the shared-MCTM. This identified two main clusters, of which the one with the most interactions included eight shared-URs (*COL1A1*, *FN1*, *SPP1*, *COL4A1*, *COL18A1*, *PLAU*, *CLEC11A*, and *MDK*) (Fig. 3C). Interestingly, seven out of eight shared-URs were more highly expressed in fibroblasts compared to other cell types in the shared-MCTM (Fig. 3D). This led us to hypothesis that fibroblasts could have a higher hierarchical role compared to other cell types in five different tumors, and that we could subtype fibroblasts to search for more genes to include in the gene signature.

### Prioritization of genes for the gene signature based on a subtype of CAF

**Fig. 4:**
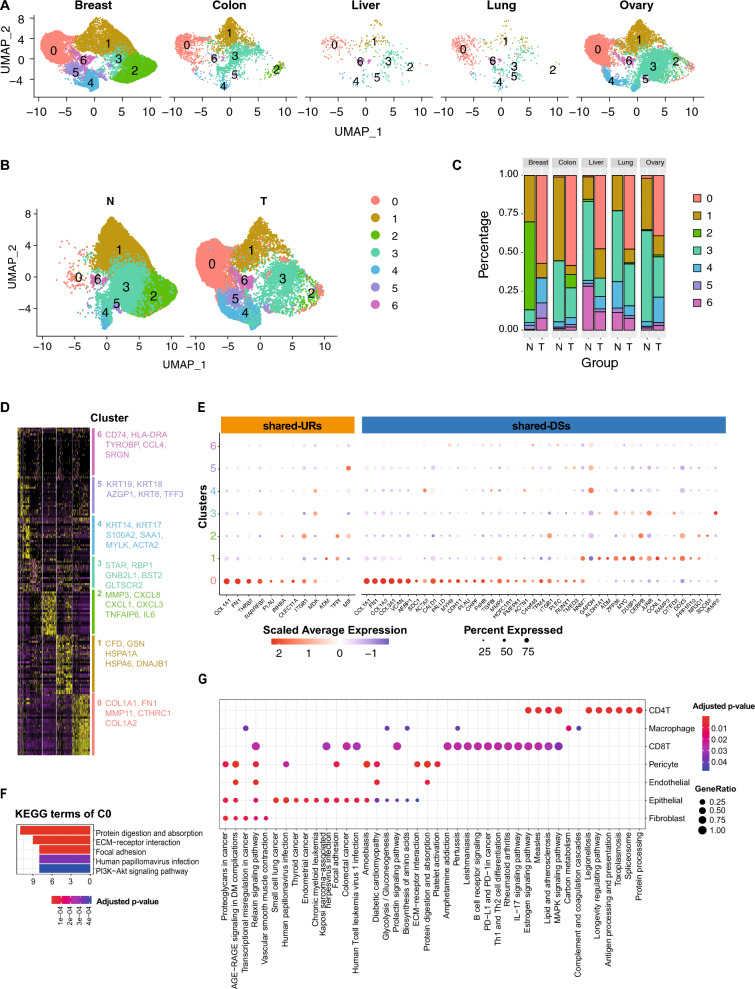
Clustering and gene expression of fibroblasts from five cancers. **A-B** Fibroblasts from all cancers were integrated; the Uniform Manifold Approximation and Projection (UMAP) shows clusters in resolution 0.1, segregated by **A** cancer type and **B** tissue type. **C** Proportions of each cluster in each cancer. **D** Heatmap showing the top marker genes for each cluster. **E** Scaled expression of fibroblast shared-URs and shared-DSs in each subcluster. The fibroblast shared-URs and shared-DSs were those that had higher expression in fibroblast compared to other cell types (log2FC > 0.25, adjusted p-value < 0.05); color scale shows the expression level while the size of dots represents the percent of cells in this cluster that expressed this gene. **F** KEGG enrichment of CAF_C0 marker genes. **G** KEGG enrichment of shared-DSs of fibroblast shared-URs. The size of each dot represents the ratio of genes mapped to each term. The number on the x-axis indicates the number of shared-DSs in each cell type. T, tumor tissue; N, adjacent normal tissue; 0–6 represents CAF clusters 0–6

To search for and prioritize subtypes of fibroblasts, fibroblasts from the five cancers were re-integrated into one dataset. A total of 36,601 fibroblast cells were clustered into seven subpopulations (Fig. 4A–C), of which four clusters (subclusters 0, 4, 5 and 6) were mainly enriched in tumor tissues, whereas subclusters 1, 2, and 3 were mainly present in normal tissues. All seven subclusters expressed canonical fibroblast markers such as *ACTA2* (*a-SMA*), while each subcluster displayed distinct transcriptomic markers (Fig. 4D and Additional Figure S4) and highly diverse functions (Additional Note 1 and Additional Figure S5). Instead of being dispersed across different fibroblast subtypes, most shared-URs and shared-DSs were highly expressed in CAF_C0 (Fig. 4E). The CAF_C0 represented the largest CAF cluster and exhibited characteristics consistent with previously reported matrix CAFs (mCAF) [4, 27], showing elevated expression of extracellular matrix (ECM) remodeling genes. Therefore, we subsequently refer to it as mCAF in the following context (Fig. 4D and F). In further support of a higher hierarchical role of mCAF, compared to other cell types, its shared-URs regulated shared-DSs in all other cell types. As commented in the discussion, KEGG pathway analysis of those shared-DSs in epithelial cells revealed a wide variety of pathways relevant for malignant transformation (Fig. 4G, Additional Figure S5 and Supplement Methods).

Taken together, the relative importance of mCAF was supported by most shared URs and DSs being expressed in this cell type, and that its shared URs regulated shared DSs in all other cell types. We therefore hypothesized that genes in mCAF could be relevant to add to the shared gene signature. For this purpose, we prioritized genes (1) with the top 10 highest log2FC between mCAF and other CAF clusters and (2) that were DEGs between tumor and normal in mCAF. This analysis resulted in eight candidate biomarkers, in addition to the eight shared-URs, namely *MMP11*, *CTHRC1*, *COL1A2*, *COL3A1*, *SPARC*, *COL5A2*, *POSTN* and *COL11A1*. In total, 16 signature genes were identified (eight shared-URs and eight mCAF marker genes).

In addition to CAF, we were also interested in whether epithelial and endothelial had subclusters that also were abundant with shared-URs and shared-DSs. However, we found no single subcluster of epithelial or endothelial cells with a predominant role similar to CAF0 (Additional Figures S6 and S7). Therefore, no signature genes were extracted from these two cell types.

### The general pathogenic relevance of the 16 signature genes and their mRNAs and protein products is supported by analyses of two large cohorts

To assess the general pathogenic relevance of these 16 signature genes, we hypothesized that the signature at both mRNA and protein levels (subsequently referred to as the mRNA signature and protein signature) (1) should be differentially expressed in tumor tissue/cancer plasma compared to normal tissue/healthy plasma and (2) associated with outcome of cancer patients—all-cause mortality in 10 years.

**Fig. 5:**
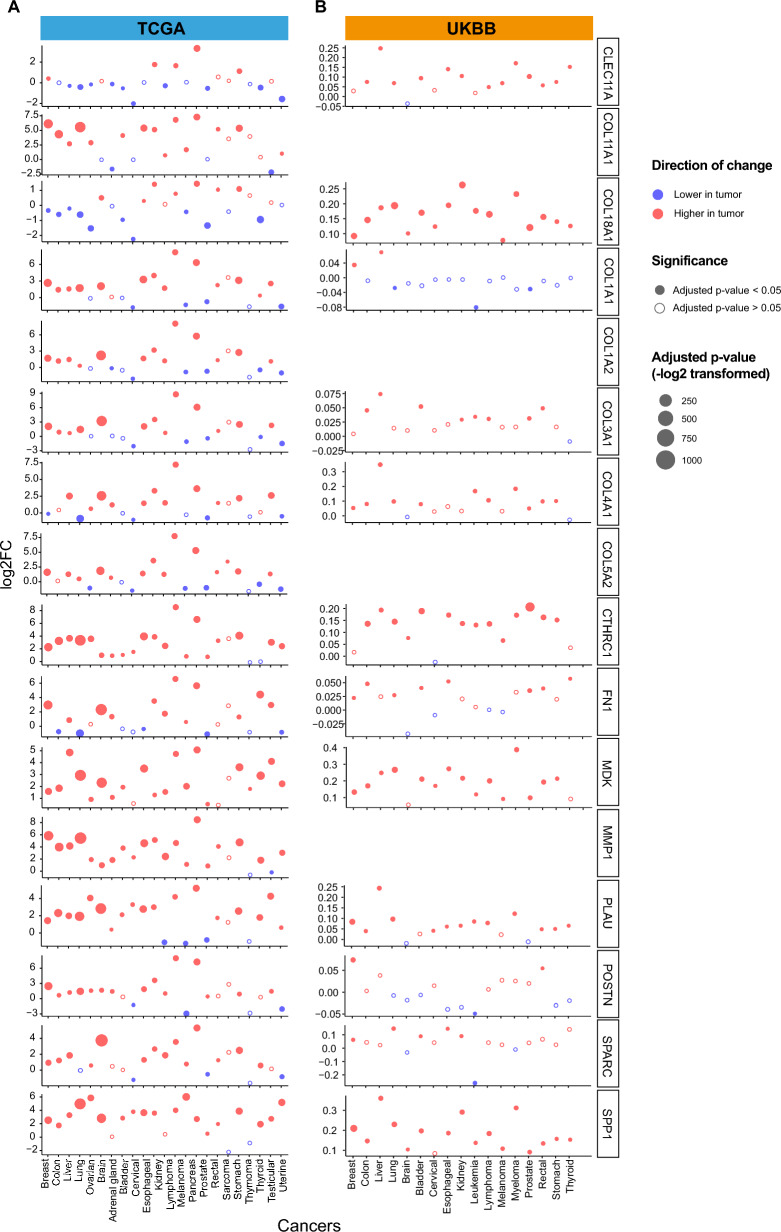
The expression difference of mRNA and protein signatures in two large cohorts. **A** The expression difference of each signature mRNA between tumor sample and normal samples in TCGA. **B** The expression difference of each signature protein in plasma between cancer patients and healthy controls in UKBB. Blue represents lower expression in tumor and red represents higher expression in tumor, filled dot means statistically significant (adjusted p-value < 0.05) while open circle means not statistically significant (adjusted p-value > 0.05). Blank area in the UKBB panel means the protein is not detected

Differential expression in tumor tissue vs. normal tissue of the mRNA signature was tested in bulk RNA sequencing data of tissue samples in TCGA (9185 patients and 727 controls from 22 cancers). The protein signatures were tested using the plasma proteomics data from the UKBB (10,384 patients and 5063 controls from 19 cancers, 12 proteins were detected) (Additional file 1 S2 to 4). These signature mRNAs/proteins were evaluated for each cancer type in both cohorts. We found that they were generally significantly differentially expressed in all cancer types on both tissue mRNA and plasma protein levels. Several mRNAs/proteins showed similar expression change in tumors from both cohorts, for example CTHRC1, MDK and SPP1, while some had more variation (e.g., COL18A1) (Fig. 5 and Additional file 6 S1 and S2). Nevertheless, the similar differential expression patterns of these signature mRNAs/proteins across different cancers suggested that this signature could represent molecular mechanisms of clinical importance. To examine this, we next analyzed if the signature was associated with one of the most important clinical trait—mortality.

### Signature genes in tumor tissues were associated with mortality in multiple cancers

**Fig. 6:**
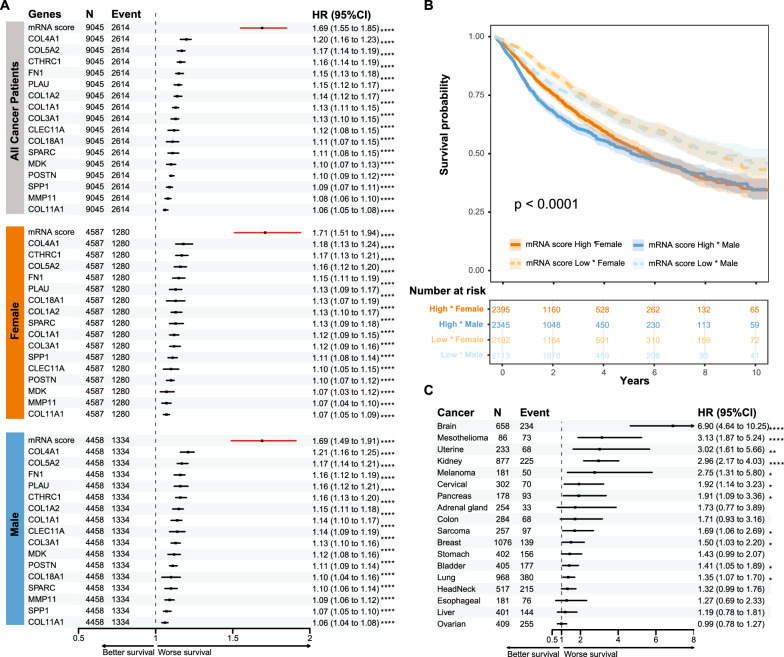
Analysis of the risk association of signature genes with 10-year mortality in TCGA cancer patients. **A** Cox regression of each signature mRNAs and the mRNA score with mortality in all cancer patients, or each sex subgroup from the TCGA cohort. **B** Cancer patients were divided into low and high score groups based on the average mRNAs score, and the Kaplan–Meier curve shows each sex and mRNAs score combination. **C** Cox regression of the gene score with mortality in each cancer type. *FDR adjusted p-value < 0.05, ** < 0.01, *** < 0.001, **** < 0.0001

The association of these signature mRNAs in tumor tissues with 10-year all-cause mortality after cancer diagnosis were evaluated using the Cox proportional hazards model in cancer patients from TCGA. The signature mRNAs showed significant associations with mortality in all cancer patients with HR ranging from 1.06 to 1.2. The mRNAs signature score was associated with higher risk of death (HR[95%CI] = 1.69[1.55–1.85]) compared to each single mRNAs (Fig. 6A and Additional file 6 S3). Similar results were found in each sex subgroup (Fig. 6A, B). When looking at each individual cancer, the mRNAs signature score was associated with mortality in 11 cancers. Particularly strong associations were found in cancers of the brain (HR[95%CI] = 6.9[4.64–10.25]), mesothelioma (HR[95%CI] = 3.13[1.87–5.24]) and uterus (HR[95%CI] = 3.02[1.61–5.66]) (Fig. 6C and Additional file 6 S4). We also repeated the analyses for another important clinical trait, namely progression free survival, and found similar results (Additional figure S8).

### Signature proteins in plasma were associated with mortality in multiple cancers

**Fig. 7:**
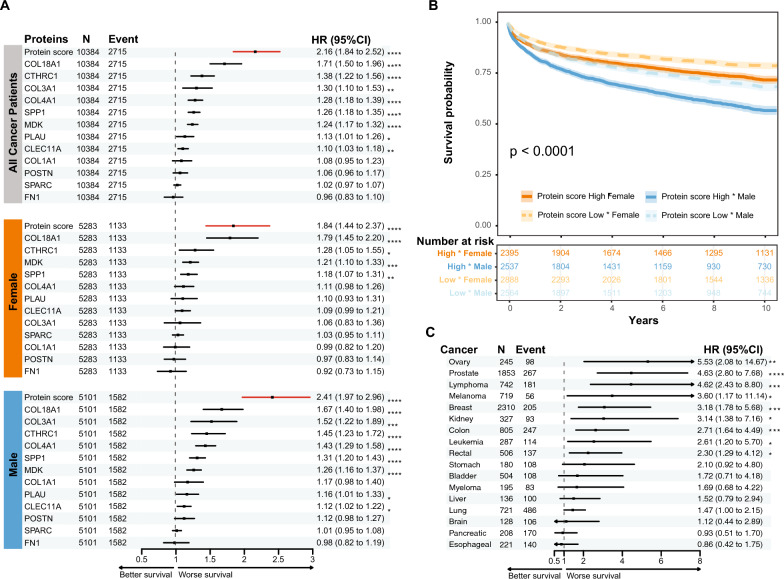
Analysis of the risk association of signature genes with 10-year mortality in UKBB cancer patients. **A** Cox regression of each signature protein and the protein score with mortality in all cancer patients, or each sex subgroup from the UKBB cohort. **B** Cancer patients were divided into low and high score groups based on the average score, and the Kaplan–Meier curve shows each sex and gene score combination. **C** Cox regression of the gene score with mortality in each cancer type. * FDR adjusted p-value < 0.05, ** < 0.01, *** < 0.001, **** < 0.0001

The association of signature proteins with survival were analyzed in plasma from cancers patients from the UKBB. A total of 12 signature proteins were identified through plasma proteomics analysis. Among these, eight plasma proteins were associated with mortality, with COL18A1 showing the highest HR in all cancer patients (HR[95%CI] = 1.72[1.92–2.50]). Compared to each individual proteins, the protein score of these nine proteins was associated with greater risk of death in all cancer patients (HR[95%CI] = 2.16[1.84,2.53]) (Fig. 7A and Additional file 6 S5). In female and male subgroups, more proteins were associated with mortality in males compared to females (8 vs. 4 proteins), while a higher protein score correlated with higher risk of death in both female and male cancer patients (Fig. 7A and B). Notably, females, overall, showed lower risk of death compared to males (Fig. 7B). The protein score of these eight proteins was associated with mortality in nine cancer types. The HR ranged from 1.47 to 5.53, with the highest HR for the death risk being found for ovarian cancer (HR[95%CI] = 5.53 [2.08–14.67]) followed by prostate cancer (4.63[2.80–7.68]) and lymphoma (HR[95%CI] = 4.62[2.43–8.8]) (Fig. 7C and Additional file 6 S6).

### Cancer type-specific signature score was not associated with mortality

While the signature derived from the shared-MCTM and mCAF showed significant association with mortality in cancer patients, we also tested if cancer type-specific signature genes could provide additional information of cancer mortality. The cancer type-specific signature genes were URs that only presented in one cancer type from the five studied scRNA-seq datasets. Cancer type-specific mRNA and a cancer type-specific protein scores were calculated for each cancer type using its cancer type-specific signature genes. Interestingly, these cancer type-specific scores were generally not associated with the mortality of the corresponding cancer type in neither TCGA nor UKBB cohorts except in liver cancer. Specifically, a higher liver-specific mRNA score was associated with improved survival among TCGA liver cancer patients (HR[95%CI] = 0.30 [0.17–0.52]) (Additional figure S9).

## Discussion

Despite the great complexity and heterogeneity of cancers this study showed molecular changes that were shared across multiple cancers. The pathogenic and clinical importance of those changes was supported by enrichment of GWAS genes and association with mortality.

The study was based on scRNA-seq, which allows the characterization of molecular changes in all cell types in a tumor. This may be advantageous because increasing evidence points to the pathogenic importance of multiple cell types in the TME [28, 29]. This complexity leads to the problems of how best to organize systematically and prioritize mechanisms across cancers.

Previous scRNA-seq studies of complex diseases, which also are multicellular, have shown that these problems can be addressed by constructing multicellular network models based on connecting URs in any cell type with their DSs in other cell types, and prioritizing the URs with the largest effects on DSs [7, 8]. We applied these principles to scRNA-seq data from five cancers. In summary, we found that despite great cellular and molecular differences among the analyzed cancers, their MCTMs showed overarching similarities. These included pathogenic URs and DSs being dispersed across cell types, rather than only originating from cancer cells. A similar organization was found in the shared-MCTM, which showed a higher-order representation of the complex changes. In support of a shared multicellular pathogenesis across cancers, the shared-MCTM was enriched for GWAS genes and pathways associated with malignant transformation. Since shared-URs regulated the several different shared-DSs, the changes in the former could have relatively greater impact than the latter. The shared-URs that regulated more shared-DSs and cells were prioritized and considered as signature genes that could have important pathogenic roles.

Notably, these prioritized shared-URs exhibited elevated expression levels in fibroblasts compared to other cell types in the shared-MCTM. This agreed with the previous finding of a hierarchy of cell–cell interactions dominated by fibroblasts to macrophages in breast cancer [5]. Moreover, we found that CAF had a potentially higher hierarchical role compared to multiple other cell types in five different tumors, supporting the crucial role of CAF in TME and tumor progression [4, 30, 31]. This led us to subtype CAF cells into clusters, of which four were more common in cancer than in normal tissues. We found that most shared-URs and shared-DSs were mainly expressed in the largest cluster (CAF_0). This cluster is in agreement with previously reported mCAF, which shows high expression of ECM remodeling genes and a pro-angiogenic effects in TME [4, 27]. Interestingly, shared-URs located in mCAF regulated shared-DSs in all other cell types. KEGG pathway analysis of those shared-DSs revealed a wide variety of pathways related to cancer, vascular function, coagulation, immunity, and metabolism. In support of a direct tumorigenic role of the fibroblast shared-URs, their shared-DSs in epithelial cells encoded cancer-related pathways, namely proteoglycan- and AGE-RAGE signaling, as well as pathways associated with many specific cancers. This finding suggested a key regulatory role of mCAF which was mainly associated with ECM according to KEGG pathway enrichment analysis. Therefore, we hypothesized that mCAF could be used to add genes to the shared gene signature. This resulted in a gene signature with eight genes from mCAF and eight shared-URs.

Recently, CCI and shared mechanisms were discussed for their potential use relates to cancer’s clinical outcomes [29, 32]. In this study, we hypothesized that this signature was associated with the mortality of cancer patients and tested the hypothesis in two independent cohorts (TCGA and UKBB). The expression of signature mRNAs and proteins showed significant differences between tumor and normal samples in both cohorts, pointing to a potential pathogenic role. Additionally, our analysis revealed that each individual signature mRNA/protein was correlated with all-cause mortality in cancer patients from both cohorts. When evaluating the overall associations of the mRNA and protein signature scores within specific cancer types, we observed moderate to high associations with mortality in both datasets. The signature genes that belong to collagen family (e.g. COL18A1 and COL4A1) showed the highest association with increased risk of death. This is in line with previous findings implicating members of the collagen family as prognostic markers for cancers [33–35]. Moreover, CTHRC1 also exhibited a high association with death risk in both mRNA and protein levels. This agrees with previous findings showing its association with tumor progression, metastasis and prognosis in several cancer types [33, 36–38]. In contrast to the above findings, our analyses of cancer-specific gene signatures did not result in significant associations with mortality in the corresponding cancer types, except for the liver-specific mRNA score. This lends further support to the pathogenic importance of shared genes and highlight the need for additional investigation into liver-specific URs.

While both mRNA and protein scores were linked to all-cause mortality, the association differed between TCGA and UKBB. The association of signature score with mortality was demonstrated to be similar between females and males in TCGA, but it was notably associated with a greater risk of death in males compared to females in the UKBB dataset. Furthermore, the cancers with the highest associations in TCGA were located in the brain, mesothelioma and uterus, while the highest associations in UKBB were ovarian cancer, prostate and lymphoma, indicating differences between tissue mRNA and blood proteins. Nevertheless, the consistent significant association of both mRNA and protein scores with mortality underscores the pathogenic relevance of the signature.

Despite this, this study has potential limitations. The shared gene expression signature was based on DEGs which are variably translated to proteins. However, one study of ovarian cancer showed that differentially expressed genes were more consistently translated to proteins than other genes [39]. To our knowledge the rates of mRNA translation between different tumors have not been systematically investigated. Another limitation is that proteins are variably released from tumor to plasma so that associations between plasma proteins and prognosis may vary. This suggests that the identified mechanisms should be carefully interpreted based on whether the source was from local tissue or blood. While the tissue mRNA signature represents mechanisms in local tumor tissue, the plasma protein signature may be derived not only from the tumor, but also from adjacent tissues and other organs. However, our analyses of plasma proteins indicate that further analyses of these proteins are warranted. Our analyses were limited to mRNAs and proteins, while multiple other types of molecules have been shown to play important pathogenic roles. Another limitation is that the scRNA-seq data were derived from a small number of patients from solid tumors. However, the relevance of the signature genes was supported in both cohorts by analyses of their associations with mortality in multiple other cancers including non-solid tumors like leukemia in independent cohorts. We propose that further studies are warranted to examine the signature genes in other cancers, as well as their associations with disease-relevant traits.

In conclusion, our findings support the pathogenic and clinical relevance of molecular interactions that are shared across cancers. We have made the methods and data underlying this study freely available for basic and translational studies (https://github.com/SDTC-CPMed/shMCTM_cancer_mortality).

### Supplementary Information

**Additional file 1: S1.** ScRNA datasets.** S2.** Data Fields and International Classification of Disease Codes Used for Identification of Cancer in the UK Biobank Cohort.** S3.** Number of patients per cancer in the UK Biobank Blood Proteomics Cohort.** S4.** Datasets from TCGA and GTEx that used for analysis.

**Additional file 2: S1.** Known Marker.** S2.** GWAS genes for each cancer from DisGeNET database.

**Additional file 3: S1.** DEGs between tumor vs. normal per cell type per cancer.** S2.** Counts of DEGs per cell type per cancer.

**Additional file 4: S1.** CCIs for breast cancer.** S2.** CCIs for colon cancer.** S3.** CCIs for liver cancer.** S4.** CCIs for colon cancer.** S5.** CCIs for ovarian cancer. **S6.** UR and DS lists for each cancer.

**Additional file 5: S1.** shared-MCTM.** S2.** GWAS enrichment for shMCTM genes.

**Additional file 6: S1.** Compare signature mRNA expression in tumor and normal in TCGA cohort.** S2.** Compare signature mRNA expression in tumor and normal in UKBB cohort. Cox results for the association of each signature gene and 10-year overall moratlity in all TCGA cancer patients. Cox results for the association of signature score and 10-year overall moratlity in each TCGA cancer. Cox results for the association of each signature gene and 10-year overall moratlity in all UKBB cancer patients. Cox results for the association of signature score and 10-year overall moratlity in each UKBB cancer.

Supplementary Material 7: Additional Figures and Notes.

Supplementary Material 8. Additional Methods.

## Data Availability

The scRNA-seq data used in this study is publicly available on GEO, with accession number GSE161529, GSE144735, GSE138709, GSE123902 and ArrayExpress E-MTAB-8107. The metadata of all the scRNA-seq datasets, URs, DSs, as well as their interactions in each dataset, and codes generated during this study are publicly available at https://github.com/SDTC-CPMed/shMCTM_cancer_mortality.

## Abbreviations

CAF: Cancer-associated fibroblast
CCI: Cell–cell interactions
CI: Confidential interval
DEG: Differentially expressed genes
DS: Downstream target
ECM: Extracellular matrix
FDR: False discovery rate
GEO: Gene expression omnibus
GTEx: Genotype-tissue expression
GWAS: Genome-wide association studies
HR: Hazards ratio
ICD: International Classification of Diseases
KNN: K-nearest neighbor
Log2FC: Log2 transformed fold change
MAST: Model-based Analysis of Single-cell Transcriptomics
MCTM: Multicellular tumor model
NPX: Normalized protein expression
scRNA-seq: Single-cell RNA sequencing
shared-MCTM: Shared multicellular tumor model
shared-DS: Shared downstream target genes
shared-UR: Shared upstream regulator gene
TCGA: The Cancer Genome Atlas
TME: Tumor microenvironment
TPM: Transcripts per million
UKBB: UK biobank
UMAP: Uniform manifold approximation and projection
UR: Upstream regulator
