# Supplementary material for: Transcript and protein signatures derived from shared molecular interactions across cancers are associated with mortality
Source: J Transl Med. 2024 May 11;22:444. doi: 10.1186/s12967-024-05268-7 (PMC11088765; doi:10.1186/s12967-024-05268-7)
Supplement: Supplementary file 7 — Supplementary Material 7: Additional Figures and Notes. [file 12967_2024_5268_MOESM7_ESM.docx]

**Additional Figures and Notes**

**Transcript and protein signatures derived from shared molecular interactions across cancers are associated with mortality**

Yelin Zhao^1^, Xinxiu Li^1^, Joseph Loscalzo^2^, Martin Smelik^1^, Oleg Sysoev^3^, Yunzhang Wang^4^, AKM Firoj Mahmud^1†^, Dina Mansour Aly^1†^, Mikael Benson^1†*^

^1^ Medical Digital Twin Research Group, Department of Clinical Science, Intervention and Technology (CLINTEC), Karolinska Institutet, Stockholm, Sweden.

^2^ Channing Division of Network Medicine, Department of Medicine, Brigham and Women’s Hospital, Harvard Medical School, Boston, Massachusetts, USA.

^3^ Division of Statistics and Machine Learning, Department of Computer and Information Science, Linköping University; Linköping, Sweden.

^4^ Department of Clinical Sciences, Danderyd Hospital, Karolinska Institutet, Stockholm, Sweden.

^†^ These contributed equally as last authors.

* Correspondence: Mikael Benson

*Corresponding author at*: Medical Digital Twin Research Group, CLINTEC, Karolinska Institute, Stockholm, Sweden.

*E-mail address*: mikael.benson@ki.se

**Additional Figures**


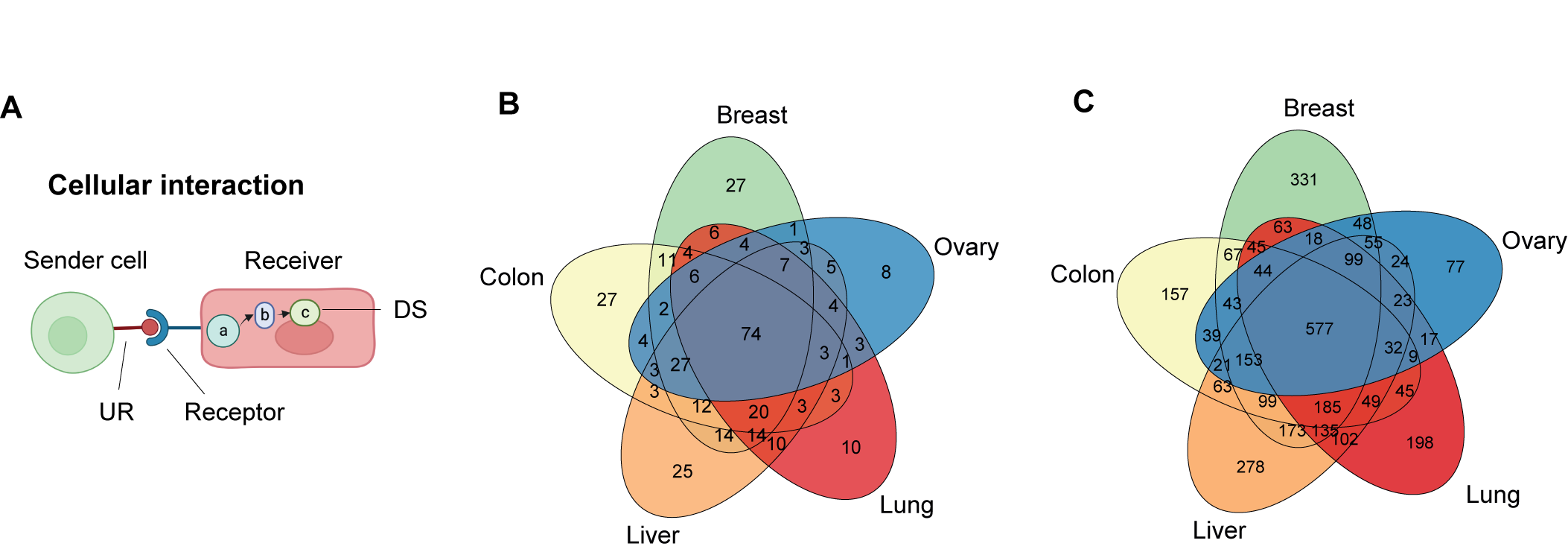


**Figure S1**. **A)** Molecular interactions between URs in any cell type and their DSs in any other cell type were predicted. **B)** The overlap of predicted URs among the five cancers and **C)** the overlap of DSs among cancers.

**Figure S2. MCTMs for each cancer.** Each dot represents a distinct cell type, with each cell type depicted in a unique color. Lines connecting dots indicate interactions, with arrows denoting the direction of interaction. The color of each line corresponds to the source cell type of the interaction. The thickness of the lines corresponds to the total number of interactions between URs in one cell type and their DSs in another cell type.


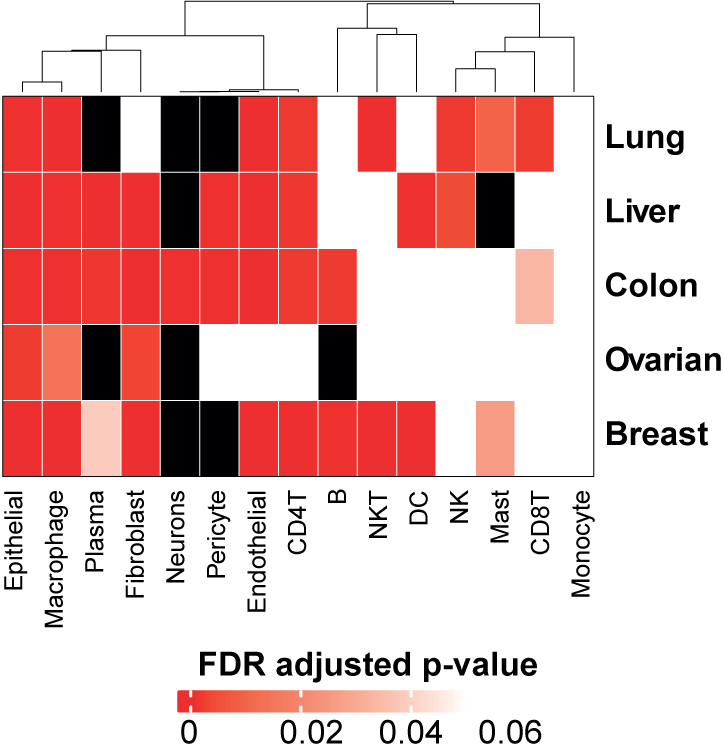


**Figure S3. GWAS gene enrichment for MCTM genes in each cell type of each cancer.** An adjusted p-value < 0.05 indicates that compared to all DEGs in each cell type/cancer, MCTM genes in this cell type/cancer significantly enriched with GWAS-associated genes. White represents enrichment adjusted p-value > 0.05, and black represents no enrichment.


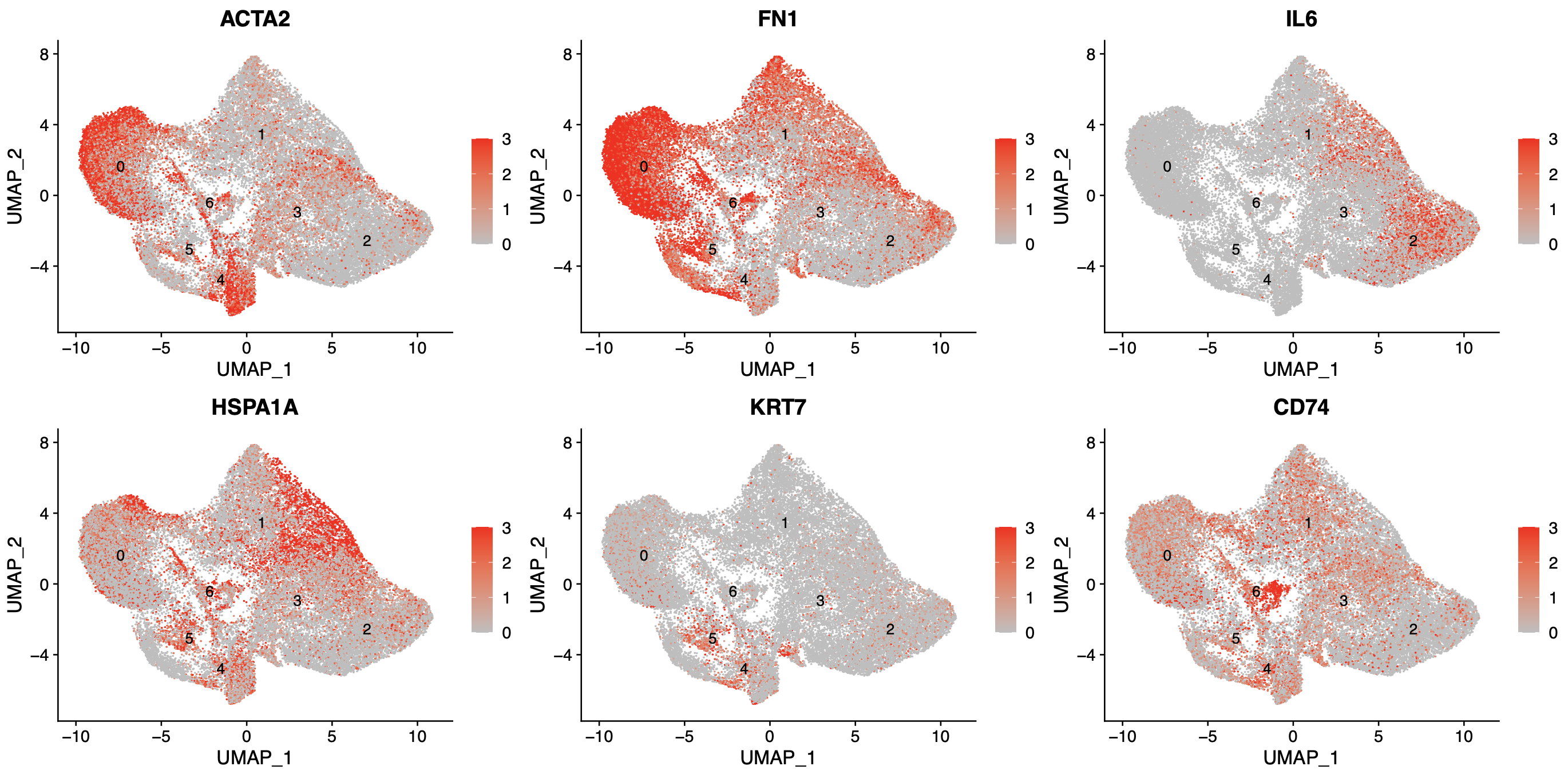


**Figure S4. Feature plot for previous known CAF clusters.** myCAF (C0 and C4): ACTA2^high^IL6^low^; iCAF (C2): ACTA2^low^IL6^high^; apCAF (C6): CD74; EMT-like CAF (C5): KRT7.

**
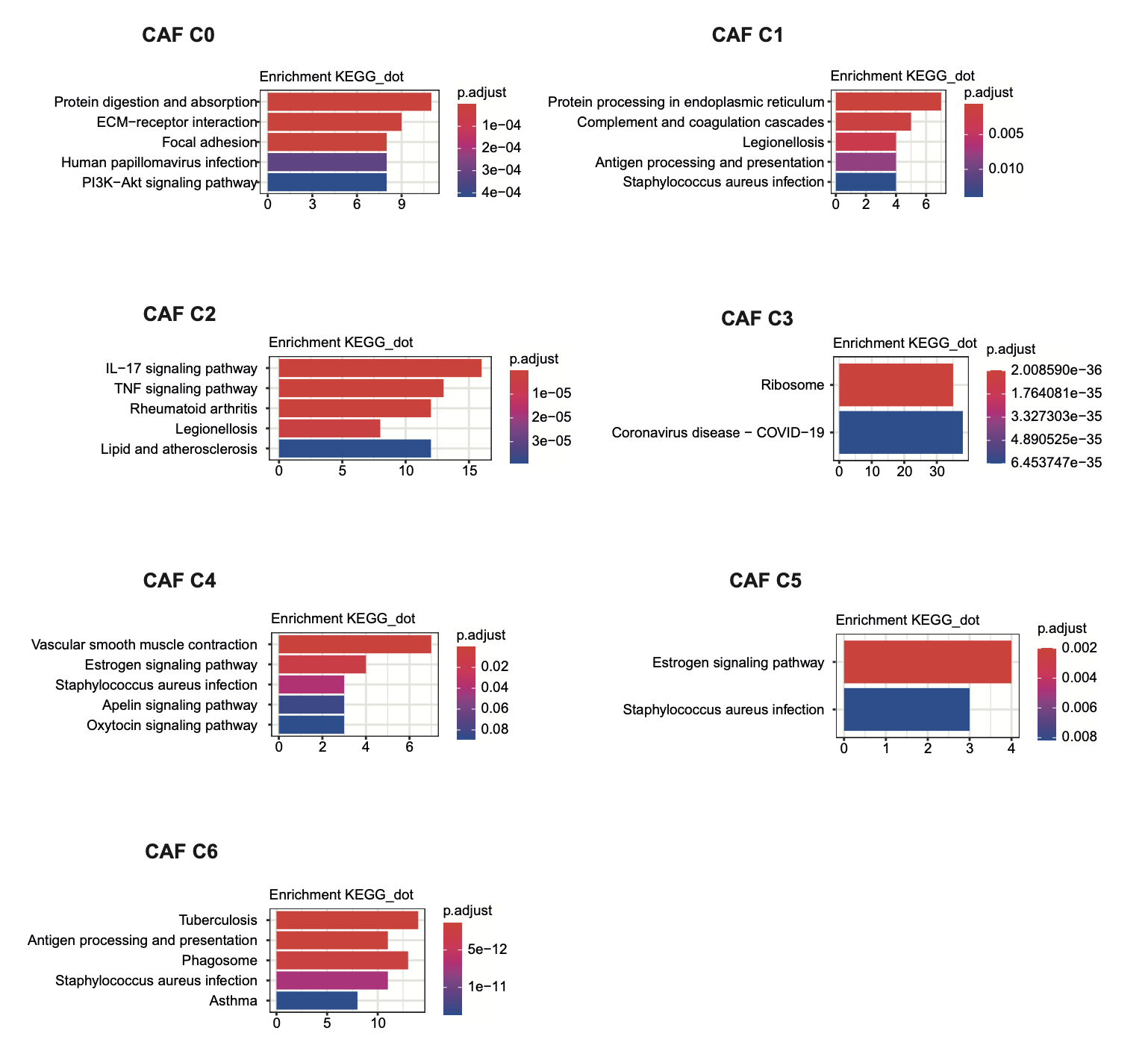
**

**Figure S5. KEGG term enrichment of CAF subclusters.** The marker genes specific to each cluster were used for KEGG term enrichment. The top five enriched KEGG terms for each cluster were presented.

**
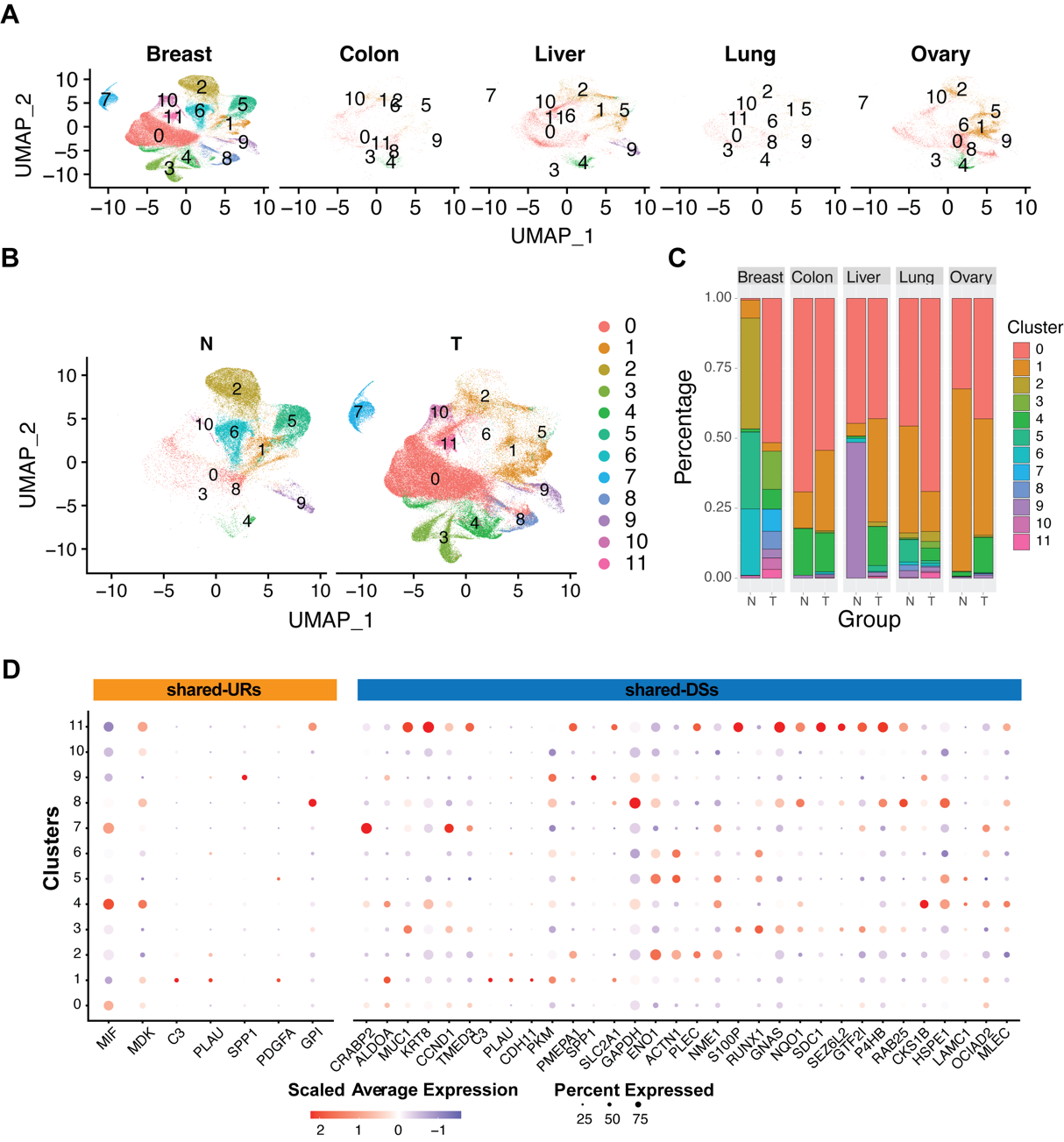
**

**Figure S6. Sub-clustering and gene expression of epithelial cells from five cancers. A-B)** Epithelial cells from all cancers were integrated; the UMAP shows clusters in resolution 0.1, segregated by **A)** cancer type and **B)** tissue type. **C)** Proportions of each cluster in each cancer. **D)** Scaled expression of epithelial shared-URs and shared-DSs in each subcluster. The epithelial shared-URs and shared-DSs were those that had higher expression in epithelial compared to other cell types (log2FC > 0.25, adjusted p-value < 0.05); color scale shows the expression level while the size of dots represents the percent of cells in this cluster that expressed this gene. T, tumor tissue; N, adjacent normal tissue; 0-11 represents clusters number.

**
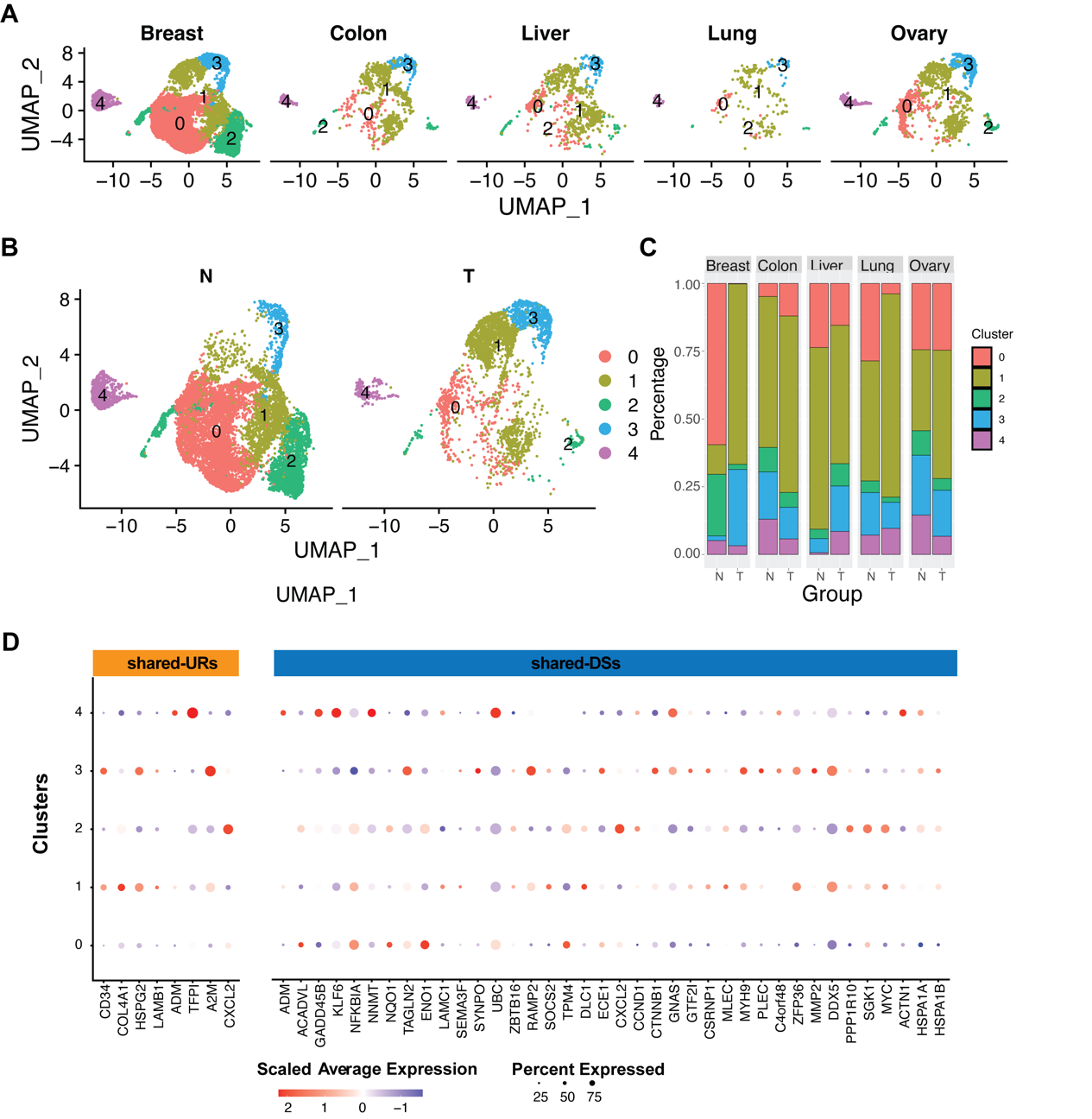
**

**Figure S7. Sub-clustering and gene expression of endothelial cells from five cancers. A-B)** Endothelial cells from all cancers were integrated; the UMAP shows clusters in resolution 0.1, segregated by **A)** cancer type and **B)** tissue type. **C)** Proportions of each cluster in each cancer. **D)** Scaled expression of endothelial shared-URs and shared-DSs in each subcluster. The endothelial shared-URs and shared-DSs were those that had higher expression in endothelial compared to other cell types (log2FC > 0.25, adjusted p-value < 0.05); color scale shows the expression level while the size of dots represents the percent of cells in this cluster that expressed this gene. T, tumor tissue; N, adjacent normal tissue; 0-4 represents clusters number.

**
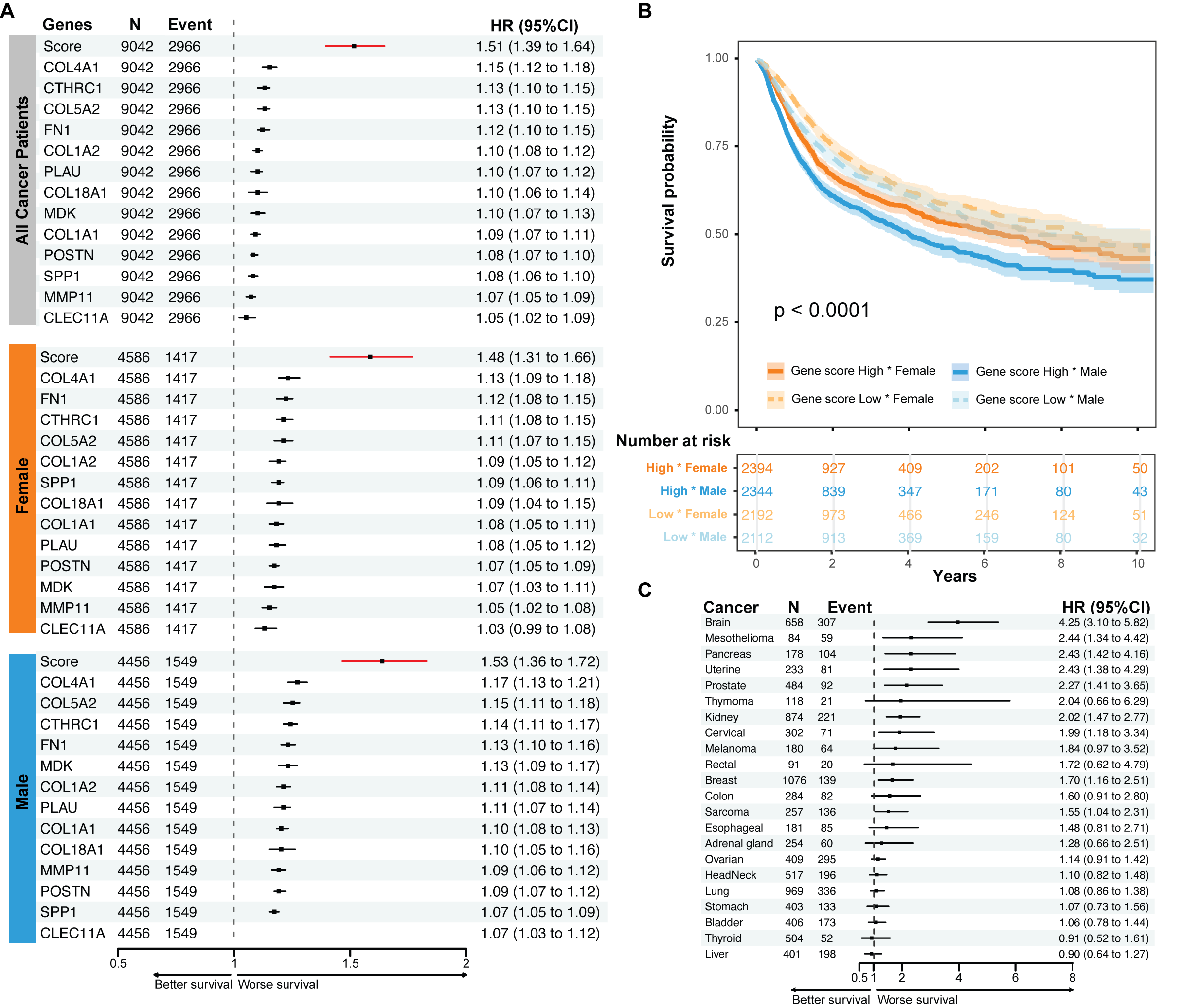
**

**Figure S8. Analysis of the risk association of signature genes with 10-year progression free mortality in TCGA cancer patients. A)** Cox regression of each signature mRNAs and the mRNA score with progression free mortality in all cancer patients, or each sex subgroup from the TCGA cohort. **B)** Cancer patients were divided into low and high score groups based on the average score, and the Kaplan-Meier curve shows each sex and gene score combination. **C)** Cox regression of the gene score with progression free mortality in each cancer type.

**
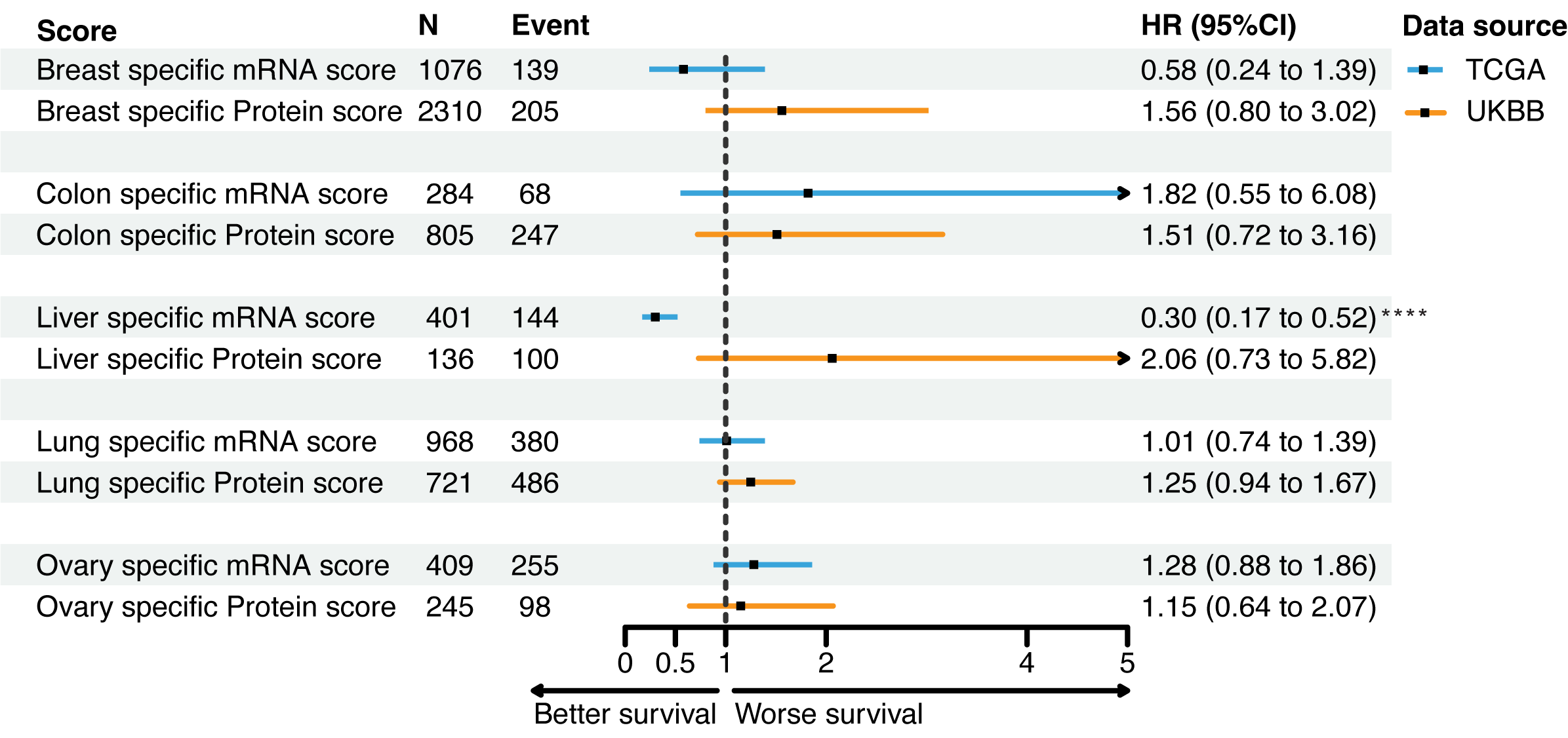
**

**Figure S9. Analysis of the risk association of cancer type-specific signature scores with 10-year mortality in TCGA and UKBB cancer patients.** Cox regression of each cancer type-specific mRNA and protein score with mortality in the corresponding cancer patients from TCGA and UKBB cohorts. Note: * FDR adjusted p-value < 0.05, ** < 0.01, *** < 0.001, **** < 0.0001.

**Supplementary Notes**

**Additional Note 1. Identification of CAF subclusters.**

All seven subclusters expressed canonical fibroblast markers such as ACTA2 (a-SMA) while each subcluster displayed distinct transcriptomic signatures (Fig. 4D) and highly diverse functions (Additional figure S5). CAF subcluster 0 was enriched with high level ACTA2 and low level of IL-6. This subcluster corresponded to known fibroblast subtype called mCAF[1]. The KEGG terms enriched for this subtype were associated with “protein digestion and absorption” and “ECM−receptor interaction”. Notably, the top signature gene of subcluster 0 (FN1 and COL1A1) were also the top shared-URs we identified in the previous step. Subcluster 1 was characterized by heat shock response proteins (HSPA1A, HSPA1B, HSPA6, DNAJB1), GSN, GPX3, IGFBP6, PI16 and complement system proteins (CFD) and was enriched in KEGG term “protein processing in endoplasmic reticulum” and “complement and coagulation cascade”. Thus, we designated this cluster as protein processing CAF (ppCAF). Subcluster 2 was classified as iCAF according to its high level of IL6 and low level of ACTA2 [2], it was enriched with inflammatory related pathways like “IL−17 signaling pathway”. Subcluster 3 was enriched in KEGG term “Ribosome” hence identified as rCAF. Subcluster 4, similar to subcluster 0, also characterized as subpopulation of myCAF (*ACTA2^high^IL6^low^*) [2]. However, it’s more related to “Vascular smooth muscle contraction” hence we define it as myCAF_v. Subcluster 5 agrees with previous known cluster EMT-like CAFs [2], it mainly expressed epithelium-specific marker genes such as KRT7, KRT8, KRT 18, and KRT19. Subcluster 6 expressed major histocompatibility complex II (MHC-II) genes such as CD74, HLA- DRA, HLA-DPA1 and HLA-DRB1, which consistent with a previous report CAF subtype of human pancreatic ductal adenocarcinoma (PDAC) [3]. We therefore termed them antigen-presenting CAFs (apCAFs) (Figure S2, S3).

1. Ma, C., et al., *Pan-cancer spatially resolved single-cell analysis reveals the crosstalk between cancer-associated fibroblasts and tumor microenvironment.* Molecular Cancer, 2023. **22**(1): p. 170.

2. Han, C., T. Liu, and R. Yin, *Biomarkers for cancer-associated fibroblasts.* Biomark Res, 2020. **8**(1): p. 64.

3. Elyada, E., et al., *Cross-Species Single-Cell Analysis of Pancreatic Ductal Adenocarcinoma Reveals Antigen-Presenting Cancer-Associated Fibroblasts.* Cancer Discov, 2019. **9**(8): p. 1102-1123.
