# Supplementary material for: Transcript and protein signatures derived from shared molecular interactions across cancers are associated with mortality
Source: J Transl Med. 2024 May 11;22:444. doi: 10.1186/s12967-024-05268-7 (PMC11088765; doi:10.1186/s12967-024-05268-7)
Supplement: Supplementary file 8 — Supplementary Material 8. Additional Methods. [file 12967_2024_5268_MOESM8_ESM.docx]

**Transcript and protein signatures derived from shared molecular interactions across cancers are associated with mortality**

Yelin Zhao^1^, Xinxiu Li^1^, Joseph Loscalzo^2^, Martin Smelik^1^, Oleg Sysoev^3^, Yunzhang Wang^4^, AKM Firoj Mahmud^1†^, Dina Mansour Aly^1†^, Mikael Benson^1†^*

^1^ Medical Digital Twin Research Group, Department of Clinical Science, Intervention and Technology (CLINTEC), Karolinska Institutet, Stockholm, Sweden.

^2^ Channing Division of Network Medicine, Department of Medicine, Brigham and Women’s Hospital, Harvard Medical School, Boston, Massachusetts, USA.

^3^ Division of Statistics and Machine Learning, Department of Computer and Information Science, Linköping University; Linköping, Sweden.

^4^ Department of Clinical Sciences, Danderyd Hospital, Karolinska Institutet, Stockholm, Sweden.

^†^ These contributed equally as last authors.

* Correspondence: Mikael Benson

*Corresponding author at*: Medical Digital Twin Research Group, CLINTEC, Karolinska Institute, Stockholm, Sweden.

*E-mail address*: mikael.benson@ki.se

# Supplementary Methods

## UK Biobank cohort plasma proteomics data

Participants of this study were a part of the UK Biobank dataset, a large prospective cohort study consisting of more than 500,000 participants recruited in the United Kingdom from 2006 to 2010. Participants were recruited at aged 40–69 years across 22 recruitment centers in the UK. Full details of the UK Biobank study can be found on the website (https://biobank.ndph.ox.ac.uk/showcase/).

A randomized subset of UK Biobank participants was conducted for the UK Biobank Pharma Proteomics Project, which proteomic profiling on blood plasma samples collected at the baseline recruitment. The second release of UK Biobank proteomics included 2,911 plasma proteins of 54,306 unique UK Biobank participants [^1^](#_ENREF_1). These proteins were tested using the antibody-based Proximity Extension Assay by Olink . The protein expression was provided as Normalized Protein Expression (NPX) values which is a relative quantification unit related to protein concentration, it was background corrected, log2 transformed and normalized within all samples[^3^](#_ENREF_3). The NPX values were downloaded and processed using the provided code 143, which allowed decoding of the data to UniProt IDs. Participants with available proteomics data were categorized into cancer group or healthy control group. The identification of cancer cases and healthy control was performed using the International Classification of Diseases (ICD) coding system, specifically ICD 9 and ICD10 (Additional file 1, S2) in data fields: “*Diagnoses - main ICD10”*, “*Diagnoses - main ICD9”*, “*Diagnoses - ICD10”*, *“Diagnoses – ICD9”*, “*Type of cancer: ICD10”* and “*Type of cancer: ICD9”*. Control samples were chosen from participants who had no illnesses or diseases code.

The UK Biobank study received ethical approval from the National Information Governance Board for Health and Social Care and the National Health Service Northwest Multi-Center Research Ethics Committee, and all participants provided written consent. This research has been conducted under approved application number 102162.

## ScRNA-seq data processing

The downloaded count matrices of each scRNA-seq data set was processed independently using the R package Seurat v4.0.4 [^6^](#_ENREF_6). For each sample, the low-quality cells were filtered based on mitochondrial RNA percentage, a consistent range of read counts, and gene coverage. At least 200 and at most 6,000 detected genes were required for each cell. Cells with exceptionally low or high numbers of reads (< 400, > 60,000) were filtered out. No more than 20% mitochondrial reads were allowed per cell [^7-10^](#_ENREF_7).

Each cancer Single-cell RNA profiles was analyzed individually. Single-cell profiles from different samples were integrated using Seurat 4 anchor-based integration methods *IntegrateData* [^6^](#_ENREF_6). The integrated data were then scaled using the *ScaleData* function. Principal component analysis (PCA) was performed on individual data sets. The optimal number of principal components was set to 30 according to the elbow plot. Cell clusters were identified using the default Louvain clustering algorithm implemented in Seurat using the *FindClusters* function. Dimensionality reduction and visualization were performed by applying the UMAP algorithm. The random seed was set to 42 to ensure reproducibility.

Cells were annotated in a unified way to ensure the comparison between cancers. Major cell types were defined by known markers: stromal cells (*PECAM1, FGF7, MME, COL1A1, ACTA2*), epithelial cells (*EPCAM, KRT7, KRT18*), myeloid cells (*CD68*), and lymphocytes (*PTPRC, CD3D, CD3E, CD8A*) and detailed subtyping were defined by known cell type marker detailed in Additional file 2.

DEGs were identified between tumor tissue and normal tissue within the same cell type in each cancer separately. A Model-based Analysis of Single-cell Transcriptomics (MAST) [^11^](#_ENREF_11) in Seurat’s *FindMarkers* function was used for DEG identification. DEGs with adjusted p-value < 0.05 and absolute log2FC > 0.25 were used for downstream analysis. For each cell type, a positive log2FC indicated upregulation in tumor tissue compared to normal tissue while a negative value indicated downregulation in tumor tissues.

**References**

1 Sun, B. B. *et al.* Plasma proteomic associations with genetics and health in the UK Biobank. *Nature* **622**, 329-338, doi:10.1038/s41586-023-06592-6 (2023).

2 Wik, L. *et al.* Proximity Extension Assay in Combination with Next-Generation Sequencing for High-throughput Proteome-wide Analysis. *Mol Cell Proteomics* **20**, 100168, doi:10.1016/j.mcpro.2021.100168 (2021).

3 UKB - Olink Explore - Data Normalization Strategy. <https://biobank.ctsu.ox.ac.uk/crystal/ukb/docs/Olink_1536_B0_to_B7_FAQ.pdf>.

4 Hanscombe, K. B., Coleman, J. R. I., Traylor, M. & Lewis, C. M. ukbtools: An R package to manage and query UK Biobank data. *PLoS One* **14**, e0214311, doi:10.1371/journal.pone.0214311 (2019).

5 Goldman, M. J. *et al.* Visualizing and interpreting cancer genomics data via the Xena platform. *Nat Biotechnol* **38**, 675-678, doi:10.1038/s41587-020-0546-8 (2020).

6 Stuart, T. *et al.* Comprehensive Integration of Single-Cell Data. *Cell* **177**, 1888-1902 e1821, doi:10.1016/j.cell.2019.05.031 (2019).

7 Pal, B. *et al.* A single-cell RNA expression atlas of normal, preneoplastic and tumorigenic states in the human breast. *EMBO J*, e107333, doi:10.15252/embj.2020107333 (2021).

8 Lee, H. O. *et al.* Lineage-dependent gene expression programs influence the immune landscape of colorectal cancer. *Nat Genet* **52**, 594-603, doi:10.1038/s41588-020-0636-z (2020).

9 Zhang, M. *et al.* Single-cell transcriptomic architecture and intercellular crosstalk of human intrahepatic cholangiocarcinoma. *J Hepatol* **73**, 1118-1130, doi:10.1016/j.jhep.2020.05.039 (2020).

10 Qian, J. *et al.* A pan-cancer blueprint of the heterogeneous tumor microenvironment revealed by single-cell profiling. *Cell Res* **30**, 745-762, doi:10.1038/s41422-020-0355-0 (2020).

11 Finak, G. *et al.* MAST: a flexible statistical framework for assessing transcriptional changes and characterizing heterogeneity in single-cell RNA sequencing data. *Genome Biology* **16**, 278, doi:10.1186/s13059-015-0844-5 (2015).
